# Supplementary material for: Associations between Circulating SELENOP Level and Disorders of Glucose and Lipid Metabolism: A Meta-Analysis
Source: Antioxidants (Basel). 2022 Jun 27;11(7):1263. doi: 10.3390/antiox11071263 (PMC9311835; doi:10.3390/antiox11071263)
Supplement: Supplementary file 1 [file antioxidants-11-01263-s001.zip › antioxidants-1740067-supplementary.pdf]

Data-processing formula

Before performing the meta-analysis, the extracted data were converted to appropriate forms. For the SELENOP concentration, data were expressed as the means and standard deviations (SDs). The formula for the conversion of standard error (SE) to SD is

$$SD = SE \times \sqrt{n}, \quad \text{(S1)}$$

with n being the sample size [1]. For studies reporting the medians and interquartile range (IQR), the medians were converted to means using

$$mean \approx \left(0.7 + \frac{0.39}{n}\right)\frac{q_1+q_3}{2} + \left(0.3 - \frac{0.39}{n}\right)m, \quad \text{(S2)}$$

where q_1 is the first quartile, q_3 is the third quartile, and m is the median [2]. The IQR was converted to SD using

$$SD \approx \frac{q_3 - q_1}{2\emptyset^{-1}\left(\frac{0.75n - 0.125}{n + 0.25}\right)}, \quad \text{(S3)}$$

where Φ^{-1} is the inverse of the cumulative standard normal distribution [3,4]. For data expressed as the geometric mean and SD, an online calculator (https://www.rapidtables.com/calc/math/Log_Calculator.html) was used to find the antilog with the base 10. The formula

$$r = 2\sin\left(r_s \times \frac{\pi}{6}\right), \quad \text{(S4)}$$

was used to convert the rho data reported as the Spearman correlation coefficient into the Pearson correlation coefficient (PCC) (where r is the PCC and r_s is Spearman's rho) [5]. Finally, for the original report and the converted correlation coefficient r values, the formula listed below

$$Fisher's\ Z = 0.5 \times ln\frac{1+r}{1-r}, \quad \text{(S5)}$$

was used to calculate the Fisher's Z value [6]. The summary Fisher's Z value (presented as the effect size (ES) and 95% confidence interval in the forest plot) was obtained by the inverse variance method [7]. Then, formula

$$summary\ r = \frac{e^{2Z}-1}{e^{2Z}+1}, \text{ (Z is summary Fisher's Z value)} \quad \text{(S6)}$$

was used to convert the value of summary r [6], which was used to comprehensively evaluate the correlation between SELENOP and glucose and lipid metabolism indicators. The degree of

correlation was judged by the range of absolute value: < 0.3 indicates poor correlation, 0.31–0.49 indicates moderate correlation, and ≥ 0.50 indicates strong correlation.

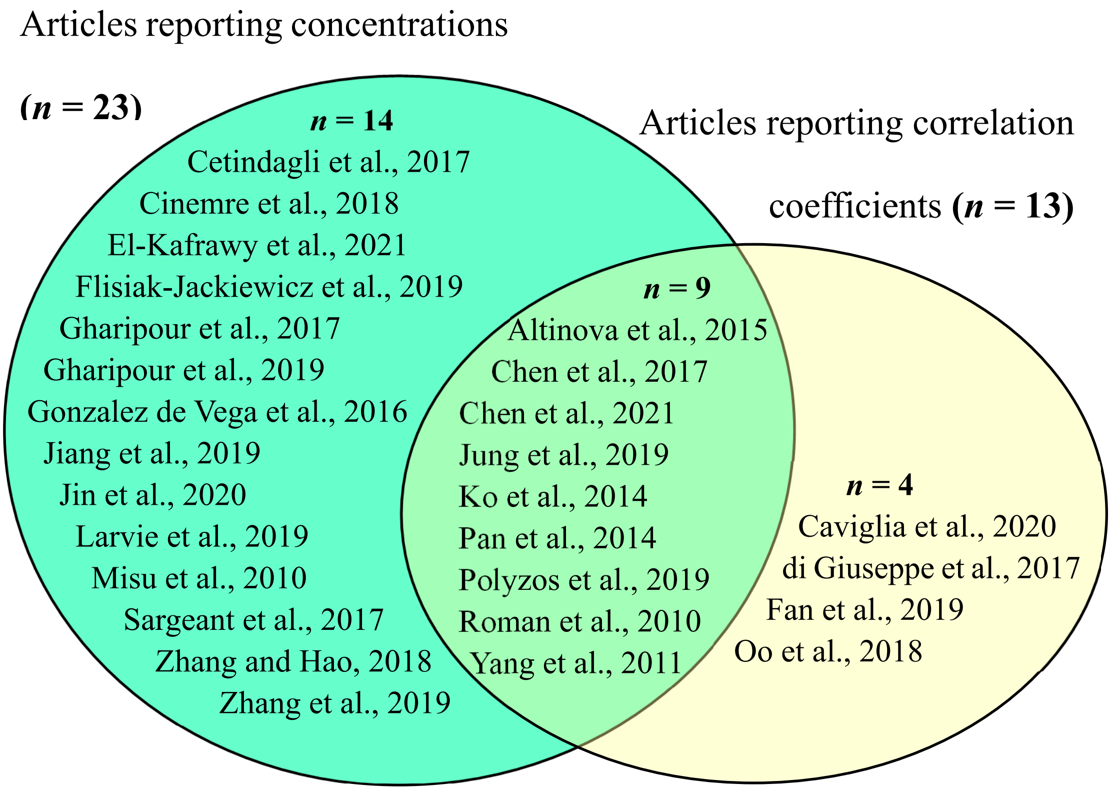

Supplementary Figure S1. Articles reporting the concentrations and/or correlation coefficients of selenoprotein P.

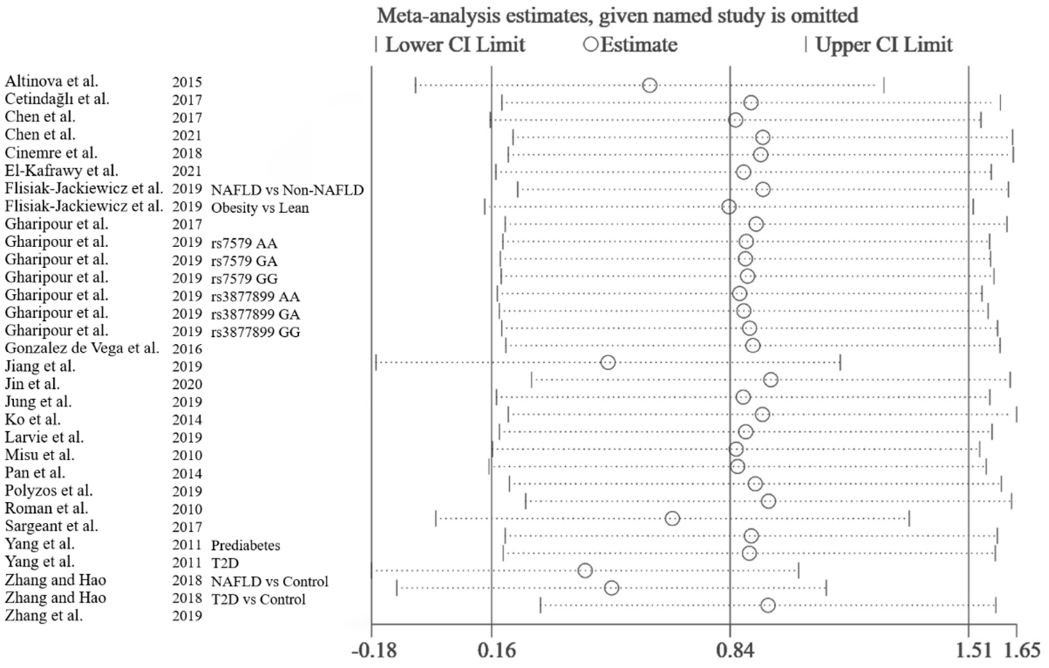

Supplementary Figure S2. Sensitivity analysis of the association between selenoprotein P and metabolic disorders of glucose and lipids. NAFLD, non-alcoholic fatty liver disease; T2D, type 2 diabetes.

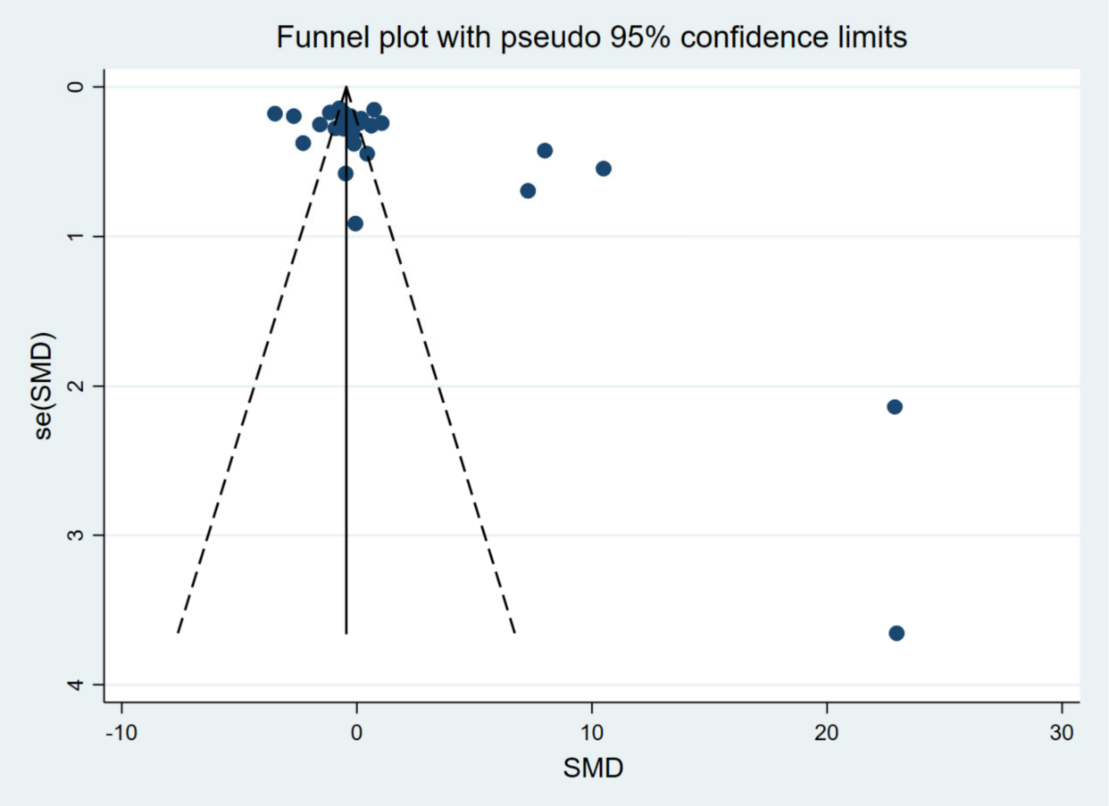

Supplementary Figure S3. Funnel plot of the meta-analysis of the association between selenoprotein P and metabolic disorders of glucose and lipids. SMD, standardized mean difference.

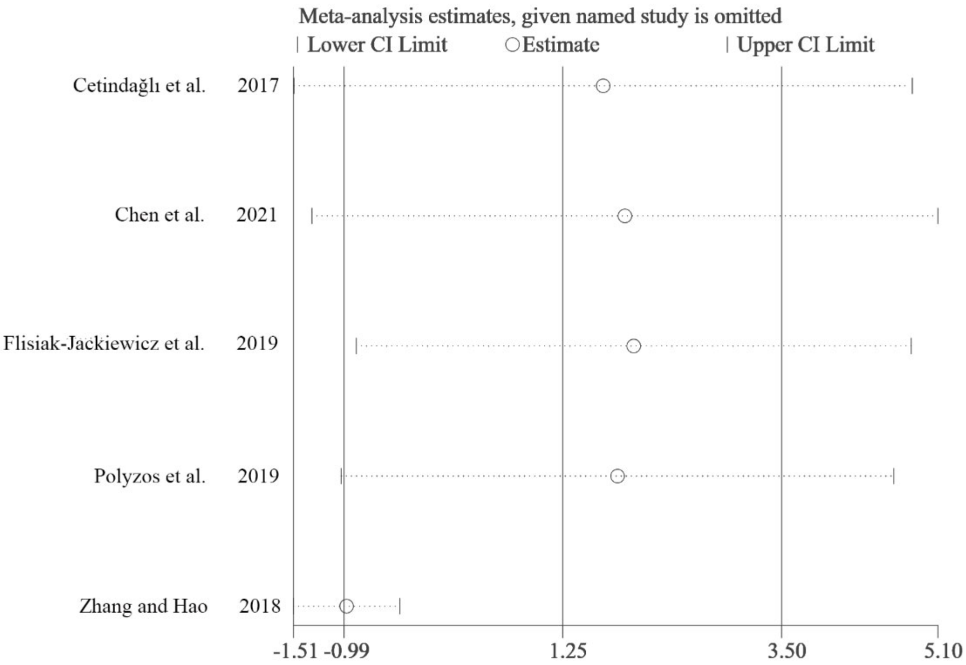

Supplementary Figure S4. Sensitivity analysis of the selenoprotein P level in non-alcoholic fatty liver disease or healthy control patients.

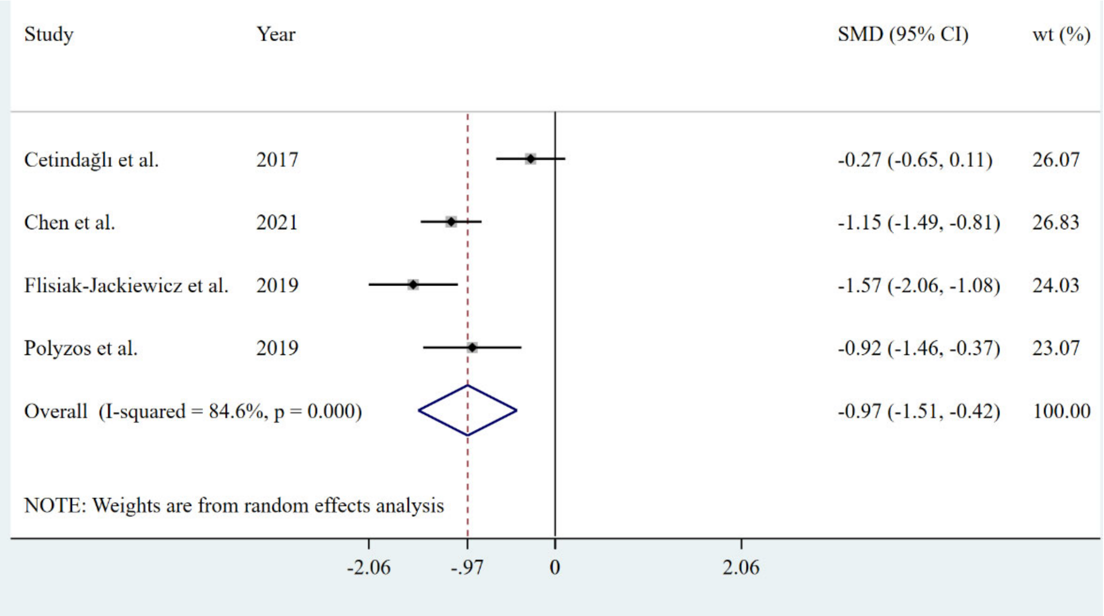

Supplementary Figure S5. Forest plot of the selenoprotein P level in non-alcoholic fatty liver disease or healthy control patients after the exclusion of one study. For each study, the length of a line parallel to the horizontal axis represents the corresponding range of the 95% CI for the adjusted SMD of the central estimate, a black diamond centered in a grey square on the line indicates the effect estimate, and the size of the grey square represents the weight. The vertical dashed line represents the summary SMD, with its 95% CI represented by the width of a hollow diamond. CI, confidence interval; SMD, standardized mean difference; wt, weight.

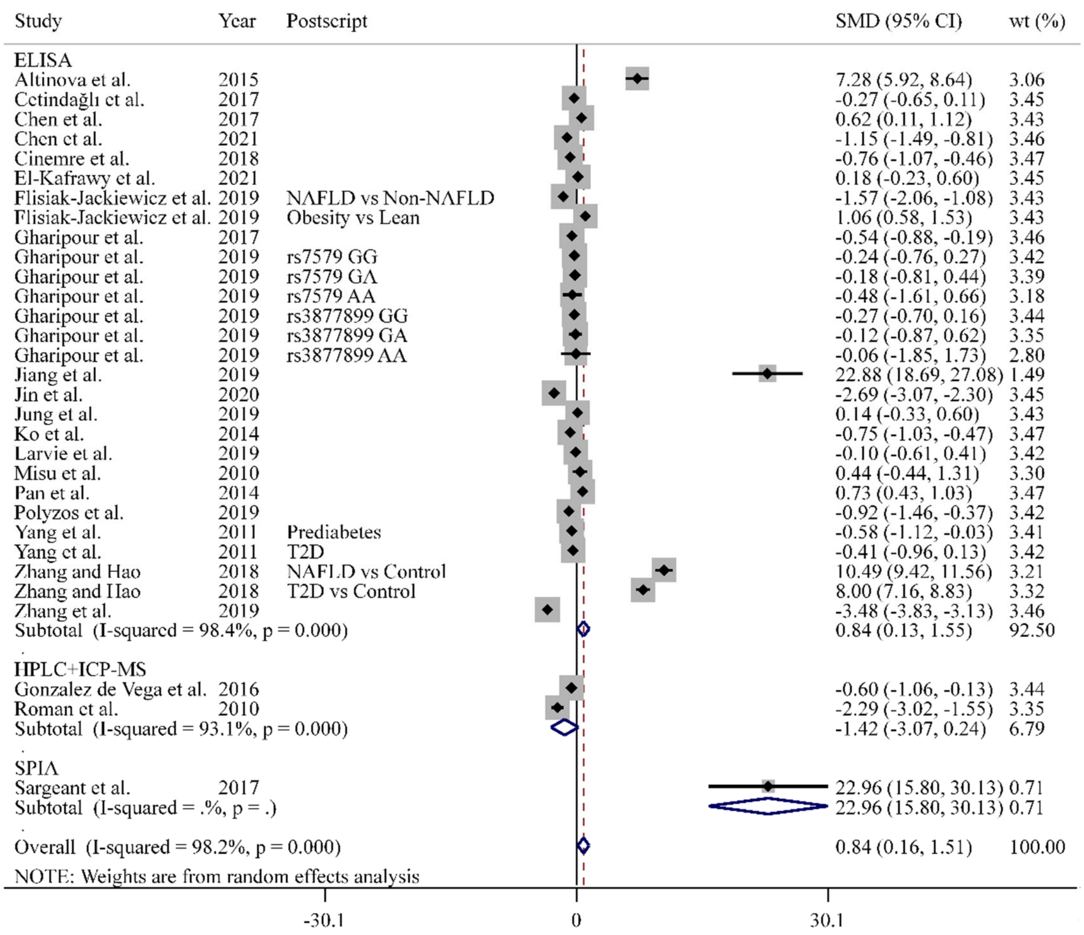

Supplementary Figure S6. Subgroup analysis based on the selenoprotein P detection method for total participants. For each study, the length of a line parallel to the horizontal axis represents the corresponding range of the 95% CI for the adjusted SMD of the central estimate, a black diamond centered in a grey square on the line indicates the effect estimate, and the size of the grey square represents the weight. The vertical dashed line represents the summary SMD with its 95% CI represented by the width of a hollow diamond. CI, confidence interval; ELISA, enzyme-linked immunosorbent assay; HPLC, high-performance liquid chromatography; ICP-MS, inductively coupled plasma-mass spectrometry; NAFLD, non-alcoholic fatty liver disease; SMD, standardized mean difference; SPIA, sol particle homogeneous immunoassay; T2D, type 2 diabetes; wt, weight.

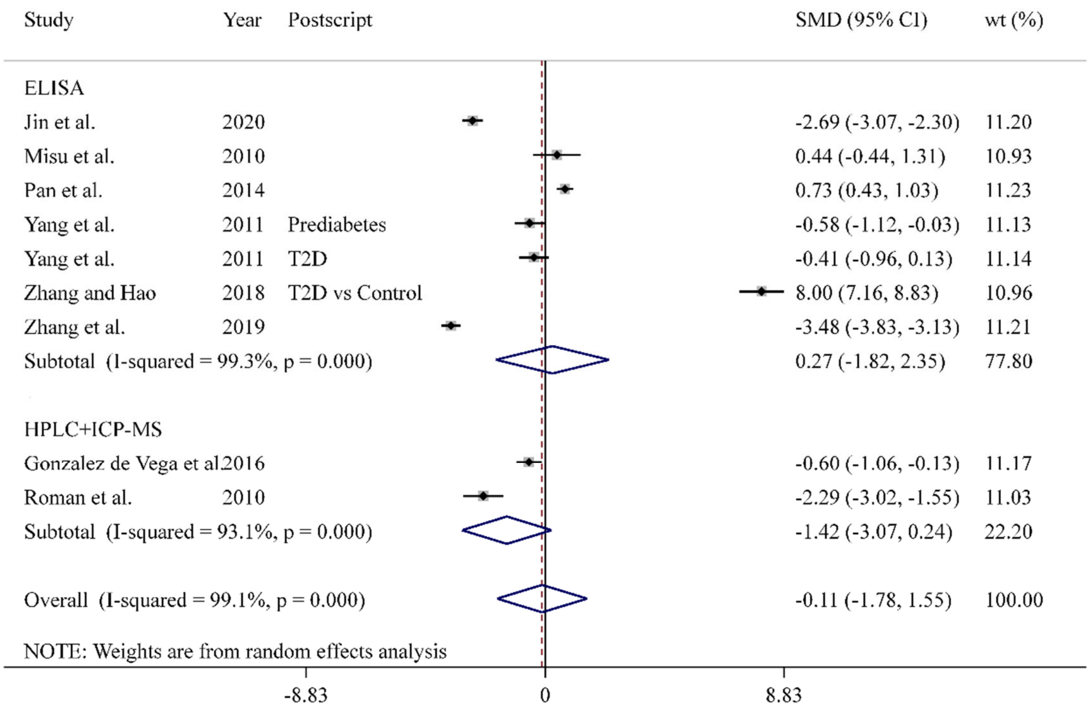

Supplementary Figure S7. Subgroup analysis based on the selenoprotein P detection method for T2D. For each study, the length of a line parallel to the horizontal axis represents the corresponding range of the 95% CI for the adjusted SMD of the central estimate, a black diamond centered in a grey square on the line indicates the effect estimate, and the size of the grey square represents the weight. The vertical dashed line represents the summary SMD with its 95% CI represented by the width of a hollow diamond. CI, confidence interval; ELISA, enzyme-linked immunosorbent assay; HPLC, high-performance liquid chromatography; ICP-MS, inductively coupled plasma-mass spectrometry; SMD, standardized mean difference; T2D, type 2 diabetes; wt, weight.

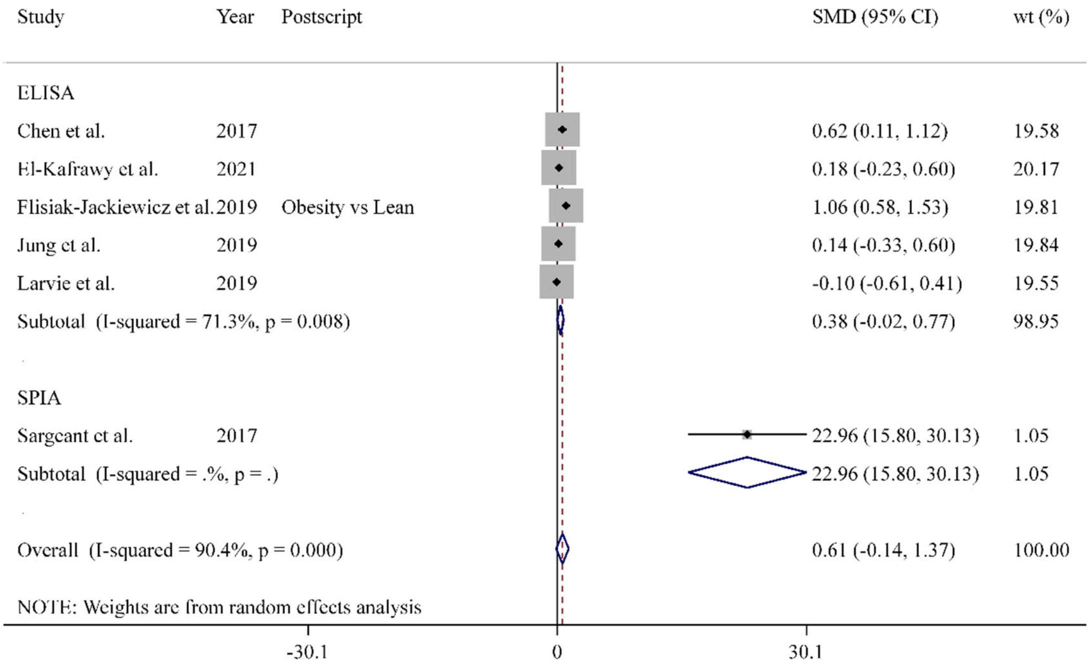

Supplementary Figure S8. Subgroup analysis based on the selenoprotein P detection method for obesity. For each study, the length of a line parallel to the horizontal axis represents the corresponding range of the 95% CI for the adjusted SMD of the central estimate, a black diamond centered in a grey square on the line indicates the effect estimate, and the size of the grey square represents the weight. The vertical dashed line represents the summary SMD with its 95% CI represented by the width of a hollow diamond. CI, confidence interval; ELISA, enzyme-linked immunosorbent assay; SMD, standardized mean difference; SPIA, sol particle homogeneous immunoassay; wt, weight.

Supplementary Table S1. Characteristics of the included studies (with indicators and correlation coefficients supplementing Table 1).

Study [a]	Disease	*n*		Level [b]		Indicator	Correlation coefficient [c]
		Case	Control	Case	Control		
*Altinova et al., 2015[8]	GD	30	35	6.2 (4.5–8.2) ♦	7.9 (4.5–10.7) ♦	HDLC	0.43€
						BMI	–0.28€
*Caviglia et al., 2020[9]	NAFLD	57		T3: 11.8		HOMA-IR	0.26€
						FPG	–0.05€
						FIns	0.28€
#Cetindağlı et al., 2017[10]	NAFLD	93	37	1574.2 ± 972.1 ♠	232.7 ± 371.05 ♠	–	–
*Chen et al., 2017[11]	OW/OB	34	29	52.3 ± 39.1 ♠	14.5 ± 12.8 ♠	TG	0.48£
						TC	0.37£
						LDLC	0.35£
						BMI	0.55£
						HOMA-IR	0.37£
						FIns	0.36£
#Chen et al., 2021[12]	NAFLD	79	79	13.4 ± 7.0 ♠	11.1 ± 7.1 ♠	TG	0.175£
						TC	0.184€
						HDLC	–0.001€
						LDLC	0.16£
						BMI	0.287£
						HOMA-IR	–0.019€
						FPG	0.15€
#Cinemre et al., 2018[13]	GD	86	90	35.29 ± 3.00 ♣	46.98 ± 4.59 ♣	–	–
*di Giuseppe et al.,	MetS	Q1: 225; Q2: 227;		Q1: 2.86 (1.96–3.70) ♦;		TG	–0.3€

Study [a]	Disease	*n*		Level [b]		Indicator	Correlation coefficient [c]
		Case	Control	Case	Control		
2017[14]		Q3: 228; Q4: 225		Q2: 4.52 (3.87–5.98) ♦;		TC	0.09[€]
				Q3: 6.05 (5.32–8.47) ♦;		HDLC	0.41[€]
				Q4: 11.72 (8.07–15.79) ♦		LDLC	–0.01[€]
						FPG	–0.15[€]
						HbA1c	–0.13[€]
*El-Kafrawy et al., 2021[15]	OW/OB	50	40	16.18 ± 3.99 ♠	4.25 ± 4.27 ♠	–	–
*Fan et al., 2019[16]	T2D and NAFLD	T2D and NAFLD: 79; T2D: 61		T2D and NAFLD: 1341.11 ± 290.51 ♠; T2D: 755.77 ± 184.90 ♠		TG	–0.149[£]
						TC	–0.106[£]
						HDLC	0.054[£]
						LDLC	0.218[£]
						BMI	0.152[£]
						HOMA-IR	0.613[£]
						FPG	0.03[£]
						FIns	0.018[£]
*Flisiak-Jackiewicz et al., 2019[17]	NAFLD	34	52	19449.5 (13327–28058) ♦	21629 (10369.5–27976) ♦	–	–
	Obesity	86	24	21421 (11566–28058) ♦	5411 (1618–15135) ♦	–	–
*Gharipour et al., 2017[18]	MetS	65	71	41.8 ± 6.57 ♣	81.5 ± 15.2 ♣	–	–
#Gharipour et al., 2019[19]	MetS	rs7579 GG: 29	30	55.52 ± 16.78 ♣	109.48 ± 29.78 ♣	–	–
		rs7579 GA: 18	22	36.65 ± 7.41 ♣	59.80 ± 22.06 ♣	–	–
		rs7579 AA: 8	5	29.45 ± 1.97 ♣	26.65 ± 2.51 ♣	–	–
		rs3877899 GG: 40	44	40.37 ± 8.44 ♣	83.91 ± 21.33 ♣	–	–
		rs3877899 GA: 15	13	56.92 ± 23.34 ♣	86.42 ± 40.99 ♣	–	–

**Study** [a]	**Disease**	***n***		**Level** [b]		**Indicator**	**Correlation coefficient** [c]
		**Case**	**Control**	**Case**	**Control**		
		rs3877899 AA: 2	3	29.70 ± 4.1 ♣	81.95 ± 107.03 ♣	–	–
#Gonzalez de Vega et al., 2016[20]	T2D	78	24	41.9 ± 12.6 ♠	50.5 ± 19.1 ♠	–	–
#Jiang et al., 2019[21]	GD	30	30	4.85 ± 1.02 ♠	2.43 ± 1.04 ♠	–	–
#Jin et al., 2020[22]	DN	100	100	673.18 ± 86.94 ♠	973.84 ± 132.27 ♠	–	–
*Jung et al., 2019[23]	OW/OB	35	35	2.3 ± 0.1 ♣	1.5 ± 0.1 ♣	BMI	0.506£
*Ko et al., 2014[24]	MetS	94	116	16.7 ± 2.2 ♥	28.6 ± 2.0 ♥	TG	–0.322£
						TC	–0.038£
						HDLC	0.217£
						BMI	–0.342£
						HOMA-IR	–0.222£
						FPG	0.038£
						FIns	–0.238£
*Larvie et al., 2019[25]	OW/OB	32	27	352.13 (276, 446) ♥	360.77 (290, 450) ♥	–	–
*Misu et al., 2010[26]	T2D	12	9	6.7 ± 0.9 ♣	5.1 ± 1.7 ♣	–	–
*Oo et al., 2018[27]	HG	76		Baseline: 2.51 ± 0.52 ♠		TG	0.004€
						BMI	–0.042€
						HOMA-IR	–0.041€
						FPG	0.194€
						HbA1c	0.113€
						FIns	–0.079€
				4-year follow-up: 3.81 ± 0.60 ♠		BMI	–0.021€
						HOMA-IR	–0.14€
						FPG	0.303€

Study [a]	Disease	n		Level [b]		Indicator	Correlation coefficient [c]
		Case	Control	Case	Control		
						HbA1c	0.164[€]
						FIns	–0.205[€]
[*]Pan et al., 2014[28]	T2D	156	64	3.77 ± 1.79 [♠]	2.34 ± 2.30 [♠]	TG	0.239[£]
						HDLC	–0.344[£]
						BMI	0.42[£]
						HOMA-IR	0.445[£]
						FPG	0.202[£]
						FIns	0.401[£]
[*]Polyzos et al., 2019[29]	NAFLD	31	27	SS: 4.2 ± 0.3 [♣];	5 ± 0.2 [♣]	BMI	–0.42[€]
				Borderline NASH: 4.1 ± 0.4 [♣];		HOMA-IR	–0.43[€]
				Definite NASH: 3.0 ± 0.5 [♣]		FPG	–0.45[€]
						FIns	–0.38[€]
[*]Roman et al., 2010[30]	T2D	40	15	58 ± 9 [♠]	56 ± 8 [♠]	TG	0.1[€]
						TC	0.07[£]
						HDLC	0.07[€]
						BMI	–0.06[€]
						FPG	0.02[€]
						HbA1c	–0.08[€]
[*]Sargeant et al., 2017[31]	OW/OB	11	11	2.81 ± 0.30 [♠]	3.01 ± 0.39 [♠]	–	–
[*]Yang et al., 2011[32]	T2D	40	20	1032.4 (495.9–2149.4) [♦];	62.0 (252.5–694.5) [♦]	TG	0.343[€]
						TC	0.07[€]
	PD	40		867.3 (516.3–1582.7) [♦]		HDLC	–0.168[€]
						LDLC	0.064[€]

Study [a]	Disease	n		Level [b]		Indicator	Correlation coefficient [c]
		Case	Control	Case	Control		
						BMI	0.259€
						HOMA-IR	0.378€
						FPG	0.335€
						HbA1c	0.264€
#Zhang and Hao, 2018[33]	T2D	100	100	3.05 ± 1.20 ♠	2.33 ± 2.30 ♠	–	–
	NAFLD	100	100	4.42 ± 1.80 ♠	2.33 ± 2.30 ♠	–	–
#Zhang et al., 2019[34]	T2D	176	142	1811.1 ± 36.3 ♣	1688.2 ± 40.5 ♣	–	–

Note: BMI, body mass index; DN, diabetic nephropathy; ELISA, enzyme-linked immunosorbent assay; FIns, fasting insulin; FPG, fasting plasma glucose; GD, gestational diabetes; HbA1c, hemoglobin A1c; HDLC, high-density lipoprotein cholesterol; HG, hyperglycemia; HOMA-IR, homeostasis model assessment of insulin resistance; HPLC, high-performance liquid chromatography; ICP-MS, inductively coupled plasma-mass spectrometry; LDLC, low-density lipoprotein cholesterol; MetS, metabolic syndrome; NAFLD, non-alcoholic fatty liver disease; NASH, non-alcoholic steatohepatitis; OW/OB, overweight and obesity; PD, prediabetes; SS: simple steatosis; SPIA, sol particle homogeneous immunoassay; T2D, type 2 diabetes; TC, total cholesterol; TG, triglyceride; n, sample size number; -, the data or information were unavailable.

[a] *, cross-sectional study; #, case-control study. [11] adjusted for BMI, age, and sex; [14] adjusted for age, sex, physical activity, education, smoking status, alcohol consumption, and C-reactive protein; [19] adjusted for age, sex, smoking, and nutrition; [24]adjusted for sex, physical activity, sleep duration, paternal education level, high-sensitivity C-reactive protein, homeostasis model assessment of insulin resistance, and alanine aminotransferase; [29] adjusted for age, sex, log (ALT), and WC; [32] adjusted for age and sex; [34] adjusted for BMI.

[b] Data were expressed as quartiles (Q1/2/3/4), tertiles (T1/2/3), medians (interquartile ranges) (♦), means ± SDs (♠), means ± SEs (♣), or geometric means ± SDs (♥) for all subjects or patients vs. controls.

[c] €, Spearman correlation coefficients; £, Pearson correlation coefficients.

Supplementary Table S2. The methodological quality evaluation of the included articles using the modified Newcastle–Ottawa scale

Article	Selection [a,b]			Comparability	Outcome [b]		Score	Quality of study
	Representativeness of samples	Sample size	Ascertainment of exposure		Assessment of the outcome	Statistical test		
Altinova et al., 2015[8]	**		√	√	√	√	9	High
Caviglia et al., 2020[9]	*		√	√[c]	√	√	7	High
Cetindağli et al., 2017[10]	*	√	√	√	√	√	9	High
Chen et al., 2017[11]	**		√	√	√	√	9	High
Chen et al., 2021[12]	**	√	√	√	√	√	10	High
Cinemre et al., 2018[13]	**	√	√	√[c]	√	√	9	High
di Giuseppe et al., 2017[14]	**	√	√	√	√	√	10	High
El-Kafrawy et al., 2021[15]	*		√	√	√	√	8	High
Fan et al., 2019[16]	*		√	√	√	√	8	High
Flisiak-Jackiewicz et al., 2019[17]	*		√	–	√	√	6	Moderate
Gharipour et al., 2017[18]	**		√	√	√	√	9	High
Gharipour et al., 2019[19]	*	√	√	√	√	√	9	High
Gonzalez de Vega et al., 2016[20]	**	√	√	–	√	√	8	High
Jiang et al., 2019[21]	*	√	√	√	√	√	9	High
Jin et al., 2020[22]	*	√	√	√	√	√	9	High
Jung et al., 2019[23]	**		√	√	√	√	9	High
Ko et al., 2014[24]	**	√	√	√	√	√	10	High
Larvie et al., 2019[25]	**	√	√	√	√	√	10	High
Misu et al., 2010[26]	*		√	√	√	√	8	High
Oo et al., 2018[27]	**	√	√	–	√	√	8	High

Article	Selection [a,b]			Comparability	Outcome [b]		Score	Quality of study
	Representativeness of samples	Sample size	Ascertainment of exposure		Assessment of the outcome	Statistical test		
Pan et al., 2014[28]	**		√	√[c]	√	√	8	High
Polyzos et al., 2019[29]	*		√	√	√	√	8	High
Roman et al., 2010[30]	*		√	√	√	√	8	High
Sargeant et al., 2017[31]		√	√	√	√	√	8	High
Yang et al., 2011[32]	**		√	√	√	√	9	High
Zhang and Hao, 2018[33]	*	√	√	√[c]	√	√	8	High
Zhang et al., 2019[34]	**	√	√	√	√	√	10	High

[a] * moderate and ** high representativeness of sample.

[b] √: Justified and satisfactory sample size, clearly ascertained exposure, comparable subjects in different outcome groups and controlled confounding factors, clearly assessed outcome, or clearly described and appropriate statistical test. - : Controlling of the confounding factors not mentioned.

[c] Only the most important factors were controlled.

References

1. Higgins JPT; Thomas J; Chandler J; Cumpston M; Li T; Page MJ; (editors)., W.V. Cochrane Handbook for Systematic Reviews of Interventions version 6.2 (updated February 2021). In *Cochrane* 2021.
2. Luo, D.; Wan, X.; Liu, J.; Tong, T. Optimally estimating the sample mean from the sample size, median, mid-range, and/or mid-quartile range. *Stat Methods Med Res* **2018**, *27*, 1785-1805, doi:10.1177/0962280216669183.
3. Wan, X.; Wang, W.; Liu, J.; Tong, T. Estimating the sample mean and standard deviation from the sample size, median, range and/or interquartile range. *BMC Med Res Methodol* **2014**, *14*, 135, doi:10.1186/1471-2288-14-135.
4. Shi J, L.D., Wan X, Liu Y, Liu J, Bian Z, Tong T. Detecting the skewness of data from the sample size and the five-number summary. *arXiv* **2020**, *2010.05749*.
5. Rupinski, M.T.; Dunlap, W.P. Approximating Pearson product-moment correlations from Kendall's Tau and Spearman's Rho. *Educ. Psychol. Meas.* **1996**, *56*, 419-429.
6. Borenstein, M.; Hedges, L.V.; Higgins, J.P.; Rothstein, H.R. A basic introduction to fixed-effect and random-effects models for meta-analysis. *Res Synth Methods* **2010**, *1*, 97-111, doi:10.1002/jrsm.12.
7. Tsiligianni, I.; Kocks, J.; Tzanakis, N.; Siafakas, N.; van der Molen, T. Factors that influence disease-specific quality of life or health status in patients with COPD: a systematic review and meta-analysis of Pearson correlations. *Prim Care Resp J* **2011**, *20*, 257-268, doi:10.4104/pcrj.2011.00029.
8. Altinova, A.E.; Iyidir, O.T.; Ozkan, C.; Ors, D.; Ozturk, M.; Gulbahar, O.; Bozkurt, N.; Toruner, F.B.; Akturk, M.; Cakir, N.; et al. Selenoprotein P is not elevated in gestational diabetes mellitus. *Gynecol. Endocrinol.* **2015**, *31*, 874-876, doi:10.3109/09513590.2015.1103220.
9. Caviglia, G.P.; Rosso, C.; Armandi, A.; Gaggini, M.; Carli, F.; Abate, M.L.; Olivero, A.; Ribaldone, D.G.; Saracco, G.M.; Gastaldelli, A.; et al. Interplay between oxidative stress and metabolic derangements in non-alcoholic fatty liver disease: the role of selenoprotein P. *Int. J. Mol. Sci.* **2020**, *21*, 1-11, doi:10.3390/ijms21228838.
10. Cetindagli, I.; Kara, M.; Tanoglu, A.; Ozalper, V.; Aribal, S.; Hancerli, Y.; Unal, M.; Ozari, O.; Hira, S.; Kaplan, M.; et al. Evaluation of endothelial dysfunction in patients with nonalcoholic fatty liver disease: association of selenoprotein P with carotid intima-media thickness and endothelium-dependent vasodilation. *Clin Res Hepatol Gastroenterol* **2017**, *41*, 516-524, doi:10.1016/j.clinre.2017.01.005.
11. Chen, M.; Liu, B.; Wilkinson, D.; Hutchison, A.T.; Thompson, C.H.; Wittert, G.A.; Heilbronn, L.K. Selenoprotein P is elevated in individuals with obesity, but is not independently associated with insulin resistance. *Obes. Res. Clin. Pract.* **2017**, *11*, 227-232, doi:10.1016/j.orcp.2016.07.004.
12. Chen, Y.; He, X.J.; Chen, X.Y.; Li, Y.M.; Ke, Y.N. SeP is elevated in NAFLD and participates in NAFLD pathogenesis through AMPK/ACC pathway. *J Cell Physiol* **2021**, *236*, 3800-3807, doi:10.1002/jcp.30121.
13. Cinemre, F.B.S.; Cinemre, H.; Yucel, A.; Degirmencioglu, S.; Tuten, A.; Yuksel, M.A.; Yilmaz, N.; Gulyasar, T.; Yildiz, M.; Bahtiyar, N.; et al. The role of selenoprotein P and selenium in the etiopathogenesis of gestational diabetes mellitus: association with

selenoprotein P1 gene (rs3877899) polymorphism. *Trace Elem Electroly* **2018**, *35*, 174-182, doi:10.5414/tex01527.
14. di Giuseppe, R.; Koch, M.; Schlesinger, S.; Borggrefe, J.; Both, M.; Müller, H.P.; Kassubek, J.; Jacobs, G.; Nöthlings, U.; Lieb, W. Circulating selenoprotein P levels in relation to MRI-derived body fat volumes, liver fat content, and metabolic disorders. *Obesity* **2017**, *25*, 1128-1135, doi:10.1002/oby.21841.
15. El-Kafrawy, N.A.F.; Atta, A.E.B.M.; Abdelsattar, S.; Zewain, S.K.E.D. Serum selenoprotein P in lean and obese Egyptian individuals and its relation to insulin resistance. *Alex J Med* **2021**, *57*, 61-69, doi:10.1080/20905068.2021.1876288.
16. Fan, H.; Shi, B.; Li, H.; Sun, H. Correlation analysis between serum selenoprotein P and non - alcoholic fatty liver disease in elders with type 2 diabetes mellitus. *Chin. J. Diabetes Mellit* **2019**, *27*, 250-253, doi:10.3969/j.issn.1006-6187.2019.04.003.
17. Flisiak-Jackiewicz, M.; Bobrus-Chociej, A.; Wasilewska, N.; Tarasow, E.; Wojtkowska, M.; Lebensztejn, D.M. Can hepatokines be regarded as novel non-invasive serum biomarkers of intrahepatic lipid content in obese children? *Adv. Med. Sci.* **2019**, *64*, 280-284, doi:10.1016/j.advms.2019.02.005.
18. Gharipour, M.; Sadeghi, M.; Salehi, M.; Behmanesh, M.; Khosravi, E.; Dianatkhah, M.; Haghjoo Javanmard, S.; Razavi, R.; Gharipour, A. Association of expression of selenoprotein P in mRNA and protein levels with metabolic syndrome in subjects with cardiovascular disease: Results of the Selenegene study. *J. Gene Med.* **2017**, *19*, doi:10.1002/jgm.2945.
19. Gharipour, M.; Ouguerram, K.; Nazih, E.; Salehi, M.; Behmanesh, M.; Razavi, R.; Gharipour, A.; Diantkhah, M.; Sadeghi, M. Effect of single nucleotide polymorphisms in SEPS1 and SEPP1 on expression in the protein level in metabolic syndrome in subjects with cardiovascular disease. *Mol. Biol. Rep* **2019**, *46*, 5685-5693, doi:10.1007/s11033-019-05000-5.
20. Gonzalez de Vega, R.; Fernandez-Sanchez, M.L.; Fernandez, J.C.; Alvarez Menendez, F.V.; Sanz-Medel, A. Selenium levels and glutathione peroxidase activity in the plasma of patients with type II diabetes mellitus. *J Trace Elem Med Biol* **2016**, *37*, 44-49, doi:10.1016/j.jtemb.2016.06.007.
21. Jiang, L.; Dou, X.; Chen, Y. Clinical analysis of serum selenoprotein P in patients with gestational diabetes mellitus. *Chin Fore Med Res* **2019**, *17*, 7-9, doi:10.14033/j.cnki.cfmr.2019.03.003.
22. Jin, S.; Jin, M.; Zhang, Y. Correlation between serum selenoprotein P and renal injury and prognosis in patients with diabetic nephropathy. *J. Trop. Med.* **2020**, *20*, 1589-1592, doi:10.3969/j.issn.1672-3619.2020.12.014.
23. Jung, T.W.; Ahn, S.H.; Shin, J.W.; Kim, H.C.; Park, E.S.; Abd El-Aty, A.M.; Hacimuftuoglu, A.; Song, K.H.; Jeong, J.H. Protectin DX ameliorates palmitate-induced hepatic insulin resistance through AMPK/SIRT1-mediated modulation of fetuin-A and SeP expression. *Clin Exp Pharmacol Physiol* **2019**, *46*, 898-909, doi:10.1111/1440-1681.13131.
24. Ko, B.J.; Kim, S.M.; Park, K.H.; Park, H.S.; Mantzoros, C.S. Levels of circulating selenoprotein P, fibroblast growth factor (FGF) 21 and FGF23 in relation to the metabolic syndrome in young children. *Int. J. Obes.* **2014**, *38*, 1497-1502, doi:10.1038/ijo.2014.45.
25. Larvie, D.Y.; Doherty, J.L.; Donati, G.L.; Armah, S.M. Relationship between selenium and hematological markers in young adults with normal weight or overweight/obesity.

Antioxidants **2019**, *8*, doi:10.3390/antiox8100463.
26. Misu, H.; Takamura, T.; Takayama, H.; Hayashi, H.; Matsuzawa-Nagata, N.; Kurita, S.; Ishikura, K.; Ando, H.; Takeshita, Y.; Ota, T.; et al. A liver-derived secretory protein, selenoprotein P, causes insulin resistance. *Cell Metab.* **2010**, *12*, 483-495, doi:10.1016/j.cmet.2010.09.015.
27. Oo, S.M.; Misu, H.; Saito, Y.; Tanaka, M.; Kato, S.; Kita, Y.; Takayama, H.; Takeshita, Y.; Kanamori, T.; Nagano, T.; et al. Serum selenoprotein P, but not selenium, predicts future hyperglycemia in a general Japanese population. *Sci Rep* **2018**, *8*, 16727, doi:10.1038/s41598-018-35067-2.
28. Pan, J.; Yu, H.; Lei, Z.; Han, J.; Jia, W. Selenoprotein P in type 2 diabetes mellitus and its association with insulin resistance. *Natl Med J Chin* **2014**, 1710-1713, doi:10.3760/cma.j.issn.0376-2491.2014.22.009.
29. Polyzos, S.A.; Kountouras, J.; Mavrouli, M.; Katsinelos, P.; Doulberis, M.; Gavana, E.; Duntas, L. Selenoprotein P in patients with nonalcoholic fatty liver disease. *Exp Clin Endocrinol Diabetes* **2019**, *127*, 598-602, doi:10.1055/a-0811-9136.
30. Roman, M.; Lapolla, A.; Jitaru, P.; Sechi, A.; Cosma, C.; Cozzi, G.; Cescon, P.; Barbante, C. Plasma selenoproteins concentrations in type 2 diabetes mellitus--a pilot study. *Transl Res* **2010**, *156*, 242-250, doi:10.1016/j.trsl.2010.07.006.
31. Sargeant, J.A.; Aithal, G.P.; Takamura, T.; Misu, H.; Takayama, H.; Douglas, J.A.; Turner, M.C.; Stensel, D.J.; Nimmo, M.A.; Webb, D.R.; et al. The influence of adiposity and acute exercise on circulating hepatokines in normal-weight and overweight/obese men. *Appl. Physiol. Nutr. Metab.* **2018**, *43*, 482-490, doi:10.1139/apnm-2017-0639.
32. Yang, S.J.; Hwang, S.Y.; Choi, H.Y.; Yoo, H.J.; Seo, J.A.; Kim, S.G.; Kim, N.H.; Baik, S.H.; Choi, D.S.; Choi, K.M. Serum selenoprotein P levels in patients with type 2 diabetes and prediabetes: Implications for insulin resistance, inflammation, and atherosclerosis. *J. Clin. Endocrinol. Metab.* **2011**, *96*, E1325-E1329, doi:10.1210/jc.2011-0620.
33. Zhang, L.; Hao, Y. Study on selenoprotein P in type 2 diabetes mellitus and non-alcoholic fatty liver disease. *J Baotou Med* **2018**, *34*, 95-96, doi:10.16833/j.cnki.jbmc.2018.09.040.
34. Zhang, Q.; Li, W.; Wang, J.; Hu, B.; Yun, H.; Guo, R.; Wang, L. Selenium levels in community dwellers with type 2 diabetes mellitus. *Biol Trace Elem Res* **2019**, *191*, 354-362, doi:10.1007/s12011-019-1645-6.
